# Screening Bioactive Compounds of Siraitia grosvenorii by Immobilized β_2_-Adrenergic Receptor Chromatography and Druggability Evaluation

**DOI:** 10.3389/fphar.2019.00915

**Published:** 2019-08-16

**Authors:** Xiaoni Jia, Jiajun Liu, Baimei Shi, Qi Liang, Juan Gao, Gangjun Feng, Zhongman Chang, Qian Li, Xiaohong Zhang, Jianbo Chen, Xinfeng Zhao

**Affiliations:** ^1^Key Laboratory of Resource Biology and Biotechnology in Western China, Ministry of Education, College of Life Sciences, Northwest University, Xi’an, China; ^2^Department of Pharmacy, Xi ‘an Mental Health Center, Xi’an, China; ^3^College of Chemistry & Chemical Engineering, Xi ‘an Shiyou University, Xi’an, China

**Keywords:** high-throughput screening, pharmacokinetics, receptor chromatography, β2-adrenergic receptor, Siraitia grosvenorii

## Abstract

As the first and key step of traditional Chinese medicine (TCM)-guided drug development, lead discovery necessitates continuous exploration of new methodology for screening bioactive compounds from TCM. This work intends to establish a strategy for rapidly recognizing β_2_-adrenergic receptor (β_2_-AR) target compounds from the fruit of *Siraitia grosvenorii* (LHG). The method involved immobilization of β_2_-AR onto amino-microsphere to synthesize the receptor column, the combination of the column to high-performance liquid chromatography (HPLC) to screen bioactive compounds of LHG, the identification of the compounds by HPLC coupled with mass spectrometry (MS), and the evaluation of druggability through pharmacokinetic examination by HPLC–MS/MS. Mogroside V was screened and identified as the β_2_-AR-targeted bioactive compounds in LHG. This compound exhibited desired pharmacokinetic behavior including the time to reach peak plasma concentrations of 45 min, the relatively low elimination of 138.5 min, and the high bioavailability. These parameters indicated that mogroside V has a good druggability for the development of new drugs fighting β_2_-AR-mediated respiratory ailments like asthma. The combination of the methods in this work is probably becoming a powerful strategy for screening and early evaluating the bioactive compounds specifically binding to G-protein-coupled receptor target from complex matrices including TCM.

## Introduction

Traditional medicine like traditional Chinese medicine (TCM) has made a great contribution to fight ailments and to protect the health of oriental people. In practice, many herbs have been prescribed as food additives in single form besides the utilization of complex prescription. Such practice necessitates the recognition of bioactive compounds not only the complex prescription but also the single herb in medicine, chemistry, and food industry (Sun et al., 2013; Bernardini et al., 2017; Zhang, 2017). This has been partly hindered for several reasons, including the complex composition, dialectical flexibility, and diverse therapeutic mechanism of TCM (Zhao et al., 2015). To address this issue, ongoing works are urgently needed to pursue the new assays that enable rapid recognition of the bioactive compounds in a single herb of complex prescription.

A survey of the literature has resulted in several modern techniques that have potential in screening bioactive compounds from TCM. These methodologies include bioactivity-guided fragmentation and separation (Sun et al., 2018; Li et al., 2019; Sari and Kececi, 2019), serum pharmacochemistry (He et al., 2015; Han et al., 2016), metabolomics (Liu et al., 2018; Wu et al., 2019), and computer virtualization technology (Mofidifar et al., 2018; Van Hilten et al., 2019). Among these assays, the bioactivity-guided fragmentation and separation are the most classic and broadly accepted method for screening compounds of interest for TCM. Despite the successful application during a long period, this method has been limited due to the loss of bioactive compounds during the gradual separating procedure. Serum pharmacochemistry has focused on identification of the compounds in serum after administration of certain TCM. Owing to such improvement, this method has the capacity to address the issue of bioactive compound loss generated by bioactivity-guided fragmentation and separation method. As a powerful method, metabolomics display an outstanding capacity to discover the biomarker of TCM and elucidate the associated mechanism. The application of this method is partly limitedly ascribed to the requirement of relatively expensive instrumental platform. Computer virtualization technology is a theoretical method that is powerful for exploration of the binding mechanism of target–compound interaction. The main issue of this method is the lack of capability in complex sample analysis. Taken together, the development of new assays for screening bioactive compounds from complex sample is still on the way to address these abovementioned issues.

Biomedical chromatography (Li et al., 2015; Wang et al., 2017a; Han et al., 2018) has the property of incorporating the specificity of target–drug recognition into the separating procedure of chromatographic analysis. It is believed to be a powerful tool for analyzing complex samples regarding the purpose of screening bioactive compounds specifically binding to the immobilized target. Although their application is successful in many cases, such methods need further improvement because most of the current methods concentrate on the transporting protein like serum albumin but much less on the largest drug targets such as G-protein-coupled receptors. Taking inspiration from the pioneer work by Wainer et al. (Wainer et al., 1999; Moaddel and Wainer, 2006; Krzysztof et al., 2010), we have developed a series of immobilized G-protein-coupled receptor-based chromatographic methods (Zhao et al., 2010; Zhao et al., 2012; Zeng et al., 2018). Broad application and validation of these methods in real sample are required to make them a powerful tool for screening bioactive compounds of TCM.

*Siraitia grosvenorii* (Swingle) C. Jeffrey ex A. M. Lu & Z. Y. Zhang is primarily cultivated in Guangxi, Guangdong, Hunan, and Jiangxi provinces of South China. The fruits of this plant, known as Luo Han Guo (LHG) in China, as a traditional medicine, have been widely used in treating coughs, sore throats, and constipation (Li et al., 2007; Qi et al., 2008; Lu et al., 2012). The mechanism of these diseases is confirmed to be associated with the signaling pathway of β_2_-adrenergic receptor (β_2_-AR) (Tamaoki et al., 2010; Cortez et al., 2016; Balabolkin, et al., 2017). Inspired by these reports, we hypothesized that *S. grosvenorii* has bioactive compounds that specifically bind to β_2_-AR. This work intends to analyze the aqueous extract of the herb by immobilized β_2_-AR-based chromatographic methods. Druggability of the screened compound was also evaluated through the analysis of pharmacokinetic behavior by high-performance liquid chromatography (HPLC) coupled with electrospray (ESI) and tandem mass spectrometry (MS/MS).

## Materials and Methods

### Materials and Instruments

**Figure 1:**
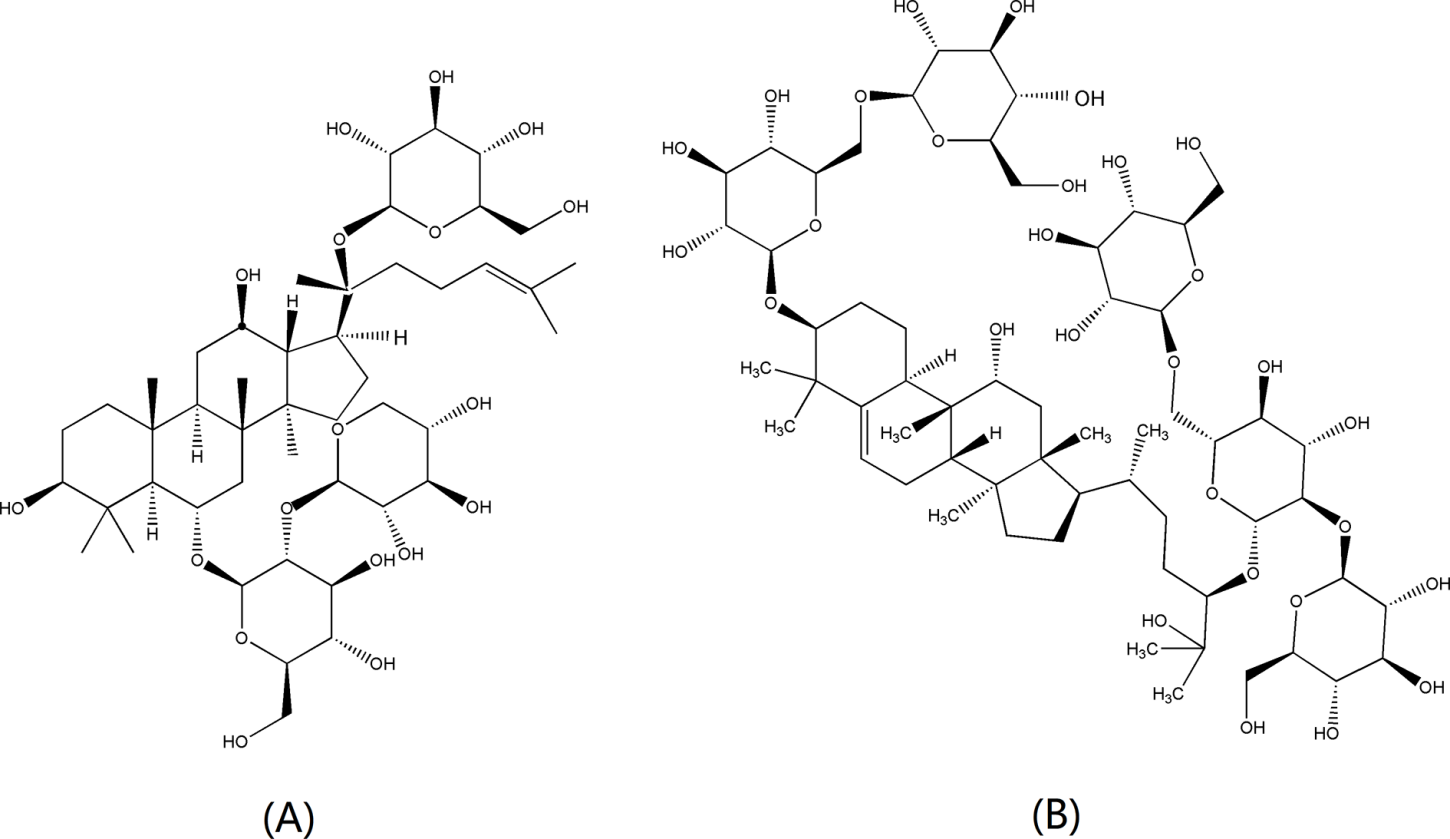
Chemical structures of** (A)** notoginsenoside R1 (IS) and **(B)** mogroside V.

Reference standards of salbutamol (batch no. 100328-200703) and terbutaline (batch no.100273-201202) were obtained from the National Institutes for Food and Drug Control (Beijing, China). Mogroside V (batch no. 181201A) was from Nanjing Daosifu Biotechnology (Nanjing, China). Notoginsenoside R1 (batch no. 110745-200415) (**Figure 1A**) was utilized as internal standard (IS) and was obtained from the National Institutes for Food and Drug Control (Beijing, China). ICI 118551 hydrochloride (batch no. 045M4622V) was bought from Sigma (St. Louis, MO, USA) and was a selective β_2_-AR antagonist. Ampicillin (Amp) was purchased from Regal Biology Technology Co (Shanghai, China). All other reagents were analytically pure unless especially stated.

The fruits of *S. grosvenorii* (LHG) were purchased from Beijing Tongrentang Herbal (Xi’an, China) and authenticated by professor Fang Minfeng, Department of Traditional Chinese Medicine of Northwest University. Voucher specimens were deposited at College of Life Sciences, Northwest University (Xi’an China), No. NWU-CLS-20181023-72. The packing machine was supplied by Dalian Elite Analytical Instruments Company (Dalian, China). The chromatographic system consisted of an Elite 3100 series apparatus (Dalian Elite Analytical Instruments Company, Dalian, China), which was equipped with an isocratic pump, a column oven, and an ultraviolet–visible detector. Separation and identification of the bioactive compounds were carried out on an Agilent 1100 high-performance liquid chromatography (HPLC) including a G1379A vacuum degasser, a G1311A quaternary pump, a G1316A thermostatted column oven, and a G1313A autosampler (Agilent, Germany) coupled with an SL Agilent 1100 tandem mass spectrometer, which was equipped with an electrospray ionization (ESI) source. *In vitro* pharmacological activity of the screened bioactive compounds was assessed by BL-420s biological function experiment system (Chengdu Techman Science and Technology Ltd, Chengdu, China). *In vitro* tissues were maintained in a physiological environment by SV-8 isothermal perfusion device (Chengdu Techman Science and Technology Ltd, Chengdu, China) and HSS-1(b) thermostatic bath (Chengdu Instrument Factory, Chengdu, China).

### Methods

#### Preparativon of LHG Extract

The aqueous extract of LHG was prepared by hot refluxing. Briefly, 100-g dried LHG was immersed in 10-folds of water and remained for 30 min. Subsequent treatment of the herb was performed by hot refluxing method with duration of 60, 50, and 40 min for each time. The suspension of each time was collected together and filtered by vacuum filtration. The filtrate was concentrated to 50 ml by rotary evaporation at 65°C. The result solution was stored at 4°C for further use.

#### Synthesis of Immobilized **β**_2_-AR Stationary Phase

According to the method in our previous work, we constructed the plasmid of pReceiver-β_2_-AR-Halo and transferred the clones into *Escherichia coli* BL21 (DE3) (Zeng et al., 2018). We incubated the clones in 50-ml Luria–Bertani (LB) medium containing 100 μg/mL of ampicillin overnight at 37°C and propagated the culture into self-induction medium for an additional 12.0-h incubation. We collected the cells by centrifugation and suspended the cell pellets in lysis buffer followed by incubation for 30 min at 37°C. This suspension was disrupted by ultrasonication and was centrifuged to collect the supernatant for immobilization.

The halo-tagged β_2_-AR protein was immobilized on the surface of amino-microspheres using the reported method (Zeng et al., 2018). Briefly, 6-chloroacetic acid-modified amino-microspheres were suspended in DMF containing *N*,*N*-diisopropylethylamine and HATU under room temperature. The suspension was stirred 4.0 h to activate the microspheres. The activated microspheres were collected by filtering the suspension and were totally rinsed by phosphate buffer (20 mM, pH = 7.4). The immobilization of β_2_-AR was achieved by mixing the activated spheres with cell lysates using phosphate buffer (20 mM, pH = 7.4) as the solvent. This suspension was stirred 2.0 h and was filtered to collect the immobilized receptor by centrifugation. The unbound protein was removed by rinsing the microspheres using phosphate buffer (20 mM, pH = 7.4). The result microspheres were packed into a stainless-steel column (50 mm × 4.6 mm) using phosphate buffer (20 mM, pH = 7.4) as slurry and propulsive agent under a pressure of 400 bar.

#### Characterization of the **β**_2_-AR Column

Sodium nitrite was used as a negative control to determine the void time of the chromatographic system. In this case, the detection wavelength was set at 254 nm. The specificity of the immobilized β_2_-AR column was characterized by retention behaviors of salbutamol and terbutaline (agonists of β_2_-AR) using ammonium acetate (5 mM, pH = 7.4) as the mobile phase. The detection wavelength was 276 nm. The stability of the column was examined by continuous analyses of the retention times and peak profiles for 3 weeks.

#### Screening of the Bioactive Compounds in LHG

Prior to screening the bioactive compounds of LHG, we analyzed the extract of the herb using reversed-phase HPLC coupled with trap mass spectrometry. Here, the separation was performed on an Inertsil ODS-3-C_18_ column (5 μm, 4.6 mm × 150 mm). The mobile phase was 0.2% (v/v) formic acid water (A) and acetonitrile (B). Gradient elution was set at 10% B to 26% B (0–5 min), 26% B (5–15 min), 26% B to 33% B (15–25 min), 33% B to 80% B (25–30 min), and 80% B to 10% B (30–35 min). The flow rate was 0.6 ml/min, and the column temperature was 30°C. MS detection was performed in a negative mode with the scan range of 100–2,000 amu. The nebulizing gas pressure was 40 psi. The flow rate and temperature of the dry gas were 8.0 L/min and 350°C, respectively.

We utilized the column containing immobilized β_2_-AR to analyze the aqueous extract of LHG. The mobile phase was ammonium acetate buffer (5 mM, pH = 7.4), and the flow rate was 0.2 ml/min with a sampling volume of 20 μl under a detection wavelength of 203 nm. The retention peak that has a retention time longer than 0.7 min was collected as the bioactive compounds specifically binding to β_2_-AR. Such collection was further separated and identified by HPLC–MS. The separating and identifying conditions for this case were identical to those in the analysis of the extract by reversed-phase HPLC–MS.

## Pharmacokinetic Study

### Animals

Male Sprague–Dawley (SD) rats (240 ± 20 g) were purchased from the Experimental Animal Center of Xi’an Jiaotong University (Xi’an, China). The animals were maintained in a room under controlled temperature (24 ± 1°C) and humidity (50–70%) with 12-h light/dark cycle. The rats were fed with standard laboratory food and acclimatized to the environment for a week before the experiment. All animals were treated in accordance with the *Guide for the Care and Use of Laboratory Animals* under the approval of the Animal Experimentation Ethics Committee at Northwest University.

### HPLC–MS/MS Conditions for Pharmacokinetic Study

All the plasma samples were analyzed on an Inertsil ODS-3-C_18_ column (5 μm, 4.6 mm × 150 mm). The mobile phase for quantitative determination of mogroside V in rat plasma consisted of 29% acetonitrile/0.2% (v/v) formic acid at a flow rate of 0.6 ml/min. The injection volume was 20 μl under a detection wavelength of 203 nm.

The detection of mogroside V in plasma sample was achieved in negative ion mode. The optimized mass conditions include the following: capillary voltage of 3,500 V, drying gas of 8 L/min at 350°C, nebulizer pressure of 40 psi at 350°C, and ion scan range of 100–2,000 amu. Under these conditions, multi-reaction monitoring (MRM) mode was carried out to specifically and sensitively detect the mogroside V. The mass transitions were *m*/*z* 1,285.6 → 1,123.6 for mogroside V and *m*/*z* 931.3 → 637.5 for IS (notoginsenoside R1).

### Preparation of Standards and Quality Control Samples

The standard stock solutions of mogroside V and IS were prepared by methanol to reach the concentration of 1.0 mg/ml. Diverse concentrations (244.1, 488.3, 976.6, 1,953.1, 7,812.5, 15,625, 31,250, and 62,500 ng/ml) of working solutions for mogroside V were prepared by diluting the stock solutions with blank plasma. A similar method was utilized to prepare the working solution of IS (50 μg/ml). All these solutions were stored at 4°C.

Calibration standards were prepared by spiking the working solutions (20 μl) into blank rat plasma (180 μl) to acquire the concentrations of 24.4, 48.8, 97.7, 195.3, 781.3, 1,562.5, 3,125, and 6,250 ng/ml. Quality control (QC) samples were prepared using the same method at 30 ng/ml (LQC), 500 ng/ml (MQC), and 5,000 ng/ml (HQC).

### Pretreatment of Plasma Sample

An aliquot of 10 μl of IS working solution was added into an aliquot of 90 μl of plasma samples. Vortexing the mixture for 2.0 min, we centrifuged the plasma for 10 min with a speed of 12,000 rpm at a temperature of 4°C. The supernatant was collected and transferred to another 0.5-ml centrifuge tube and then blow dried with nitrogen. We applied 200 μl of methanol to redissolve it, which was used for analysis.

### Validation of the Method for Pharmacokinetic Study

#### Selectivity and Specificity

The selectivity and specificity of the method were evaluated by analyzing the chromatograms of six different batches of blank plasma, blank plasma spiked with mogroside V, and IS, and the samples after administration of mogroside V.

#### Linearity of Calibration Curves and Lower Limits of Quantification

The calibration curve was obtained by the peak area ratios (*y*) of mogroside V to IS against the theoretical concentration (*x*) of mogroside V. Linearity of calibration curves was assessed by a linear regression using 1/*x* ^2^ as weighting factor. The lower limit of quantification (LLOQ) having signal/noise (S/N) ratio ≥ 10 with acceptable accuracy [relative error (RE)] and precision [relative standard deviation (RSD)] of less than 20% was defined as the lowest drug concentration of the calibration curve.

#### Accuracy and Precision

Accuracy and precision were evaluated at three QC levels in six replicates on the same day and three analytical batches on six continuous days. Intra-day and inter-day precision [relative standard deviation (RSD)] and the accuracy [relative error (RE)] of such cases were required to be less than ±15%.

#### Extraction Recovery

The extraction recoveries of the analytes were determined at three levels of QC samples. They were calculated by comparing the mean peak area of QC samples with those of the blank plasma spiked with neat solutions after extraction (*n* = 6).

#### Stability

The stability of mogroside V in plasma was evaluated at three QC levels in six replicates under varieties of storing and processing conditions. The results of the peak area of QC samples were compared with those from freshly prepared standard samples. The QC samples with recoveries of 85–115% were defined as having good stability under the desired conditions. Short-term stability was assessed after QC samples were saved at room temperature for 14 h. Subsequently, freeze–thaw stability was assessed after three freeze (−20°C) and thaw (room temperature) cycles of QC samples. Long-term stability was examined after QC samples were saved at −20°C for 1 month.

### Pharmacokinetic Study of Mogroside V

Eighteen rats were randomly divided into three groups of six for each group, including blank control, mogroside V, and LHG groups. The blank group was orally administered with normal saline. The other two groups were administered with mogroside V at a dose of 200 mg/kg and LHG aqueous extraction at a dose of 12.5 ml/kg (equivalent to 200 mg/kg mogroside V). Blood samples were collected in heparin sodium vacutainers at post-dose 5, 15, 30, 45, 60, 90, 120, 240, 360, 480, and 720 min. The plasma was pre-treated by centrifugation and frozen at −80°C until further analysis. The pharmacokinetic parameters including the area under curve (AUC_0–720_, and AUC_0–∞_), the maximum plasma concentration (C_max_), the time to achieve maximum plasma concentration (*T*_max_), and the elimination half-life (*t*_1/2z_) were calculated by Drug and Statistics 3.0.0 software (DAS, T. C. M., Shanghai, China). All data were recorded as the mean ± standard deviation (SD), and the 95% confidence interval was also estimated when necessary.

## Results and Discussion

### Feasibility of Immobilized β_2_-AR in Screening Bioactive Compounds of LHG

It is reported that the aqueous extract of LHG has significant inhibitory effect on cough of mice induced by concentrated ammonium hydroxide or sulfur dioxide. The extract is also attractive for increasing the secretion function of the respiratory tract and has a laxative effect on normal or constipated mice and anti-spasmolytic effect on isolated small intestine (Wang et al., 1998). Other publications have showed that the aqueous extract of LHG and *Momordica* glycosides could significantly reduce the cough numbers and increase the amount of tracheal secretions in cavy and mice, respectively (Chen et al., 2006; Li et al., 2008). As one of the effective components of LHG, mogroside V (**Figure 1B**) is extensively discussed for reducing the cough times and prolonged the incubation period for cough of mice (Liu et al., 2007). It is also effective for extending the phenol red excretion of mice trachea, which indicates that mogroside V has a certain expectorant effect. In addition, mogroside V has obvious capability to antagonize spasmodic contraction of the ileum and trachea caused by histamine. These results have indicated that mogroside V has certain contribution to the antitussive, expectorant, and spasmolytic effects of LHG.

In a pharmacological view, these ailments are closely associated with the signaling pathway of β_2_-AR. For instance, the work by Freund-Michel et al. (2010) has declared that β_2_-AR agonists exhibit antitussive properties in guinea pigs through direct inhibition of sensory nerve activity, independent of bronchodilation. These aforementioned reports have demonstrated that there are bioactive compounds targeting β_2_-AR in LHG extract; mogroside V is possible to become a ligand of β_2_-AR. Regarding these demonstrations, we proposed a good feasibility of immobilized β_2_-AR in screening bioactive compounds binding to the receptor from LHG extract.

### Analysis of LHG Extract by RP–HPLC–MS/MS

*S. grosvenorii* is a medicinal and edible plant that belongs to *Cucurbitaceae* vine family. It contains diverse bioactive ingredients, such as triterpenoids, flavonoids, lignans, furans, sugars, fatty acids, and steroid ingredients. Previous reports have isolated and identified nearly 50 kinds of triterpenoids, seven kinds of flavonoids, and 59 kinds of fatty acids from the herb (Chaturvedula and Prakash, 2011; Chaturvedula and Meneni, 2015; Takemoto et al., 1983a, b, c; Qing et al., 2017). Takemoto et al. (1983a), Takemoto et al. (1983b) and Takemoto et al. (1983c) have confirmed mogroside IV, mogroside V, and mogroside VI isolated from *S. grosvenorii* as tetracyclic triterpene compound that exhibits a wide range of notable biological activities. As a member of the triterpene glycosides, mogrosides are regarded as the major effective constituents of *S. grosvenorii*. Afterward, a large group of triterpene glycosides were identified from dried and fresh fruits of the herb. These compounds include mogroside IV, mogroside V, mogroside III, 11-oxidized-mogrol V, mogroside IE_1_, mogroside IIIE, siamenoside I, mogroside IVa, mogroside IIA_1_, mogroside IIA_2_, glycoside VI, mogroside A, isomogroside V, and mogroside IIE (Chaturvedula and Prakash, 2011; Prakash and Chaturvedula, 2014; Qing et al., 2017).

**Figure 2:**
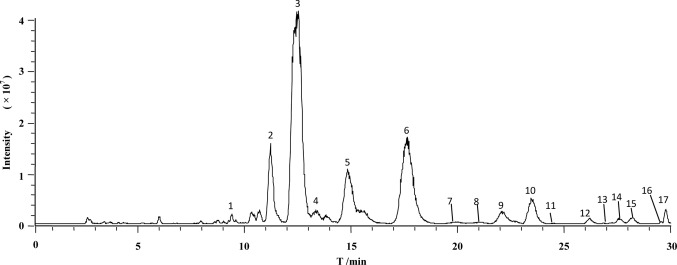
Representative chromatogram of total ion current of LHG extract by reversed-phase high-performance liquid chromatography coupled with mass spectrometry.

**Figure 2** illustrates the total ion current chromatogram of the aqueous extract. With reference to those reports, we identified 17 compounds in the herb. Among them, 12 were triterpene saponins, and 5 were not yet identified to our scope of knowledge (**Table 1**). Additionally, the content of mogroside V was determined as 0.8% of *S. grosvenorii*, which is much higher than the value of 0.5% required by Chinese Pharmacopoeia 2015 Edition. This result confirmed the quality of *S. grosvenorii* collected in this work.

**Table 1:** MS parameters for analysis of LHG extract.

No.	t_R_ (min)	Analytes	Molecular formula	m/z precursor
1	9.3	Grosvenorine^a^	C_33_H_40_O_19_	[M−H]^−^ 739.2
2	11.1	11-Oxo-mogroside V^a^	C_60_H_99_O_29_	[M−H]^−^ 1,283.8
3	12.4	Mogroside V^a^	C_60_H_101_O_29_	[M−H]^−^ 1,285.6
2	13.3	Isomogroside V^a^	C_60_H_101_O_29_	[M−H]^−^ 1,285.6
5	14.7	Siamenoside I^a^	C_54_H_92_O_24_	[M−H]^−^ 1,123.7
6	17.6	Mogroside IVa^a^	C_54_H_92_O_24_	[M−H]^−^ 1,123.8
7	19.9	Mogroside IVe^a^	C_54_H_92_O_24_	[M−H]^−^ 1,123.6
8	21	Mogroside III^a^	C_48_H_82_O_19_	[M−H]^−^ 961.5
9	22	Mogroside IIIe^a^	C_48_H_82_O_19_	[M−H]^−^ 961.6
10	23.4	Unknown	Unknown	[M−H]^−^ 1,269.8
11	24.8	Mogroside IIIA_1_^a^	C_48_H_82_O_19_	[M−H]^−^ 961.5
12	26.1	Unknown	Unknown	[M−H]^−^ 1,269.8
13	26.8	Unknown	Unknown	[M−H]^−^ 1,269.8
14	27.5	Unknown	Unknown	[M−H]^−^ 1,107.6
15	28.2	Mogroside IIIA_2_^a^	C_48_H_82_O_19_	[M−H]^−^ 961.6
16	29.5	Unknown	Unknown	[M−H]^−^ 1,107.6
17	29.7	Mogroside IIA_2_^a^	C_42_H_72_O_14_	[M−H]^−^ 845.6

^a^Is reference to Chaturvedula and Prakash, 2011.

### Characterization of β_2_-AR Column

**Figure 3:**
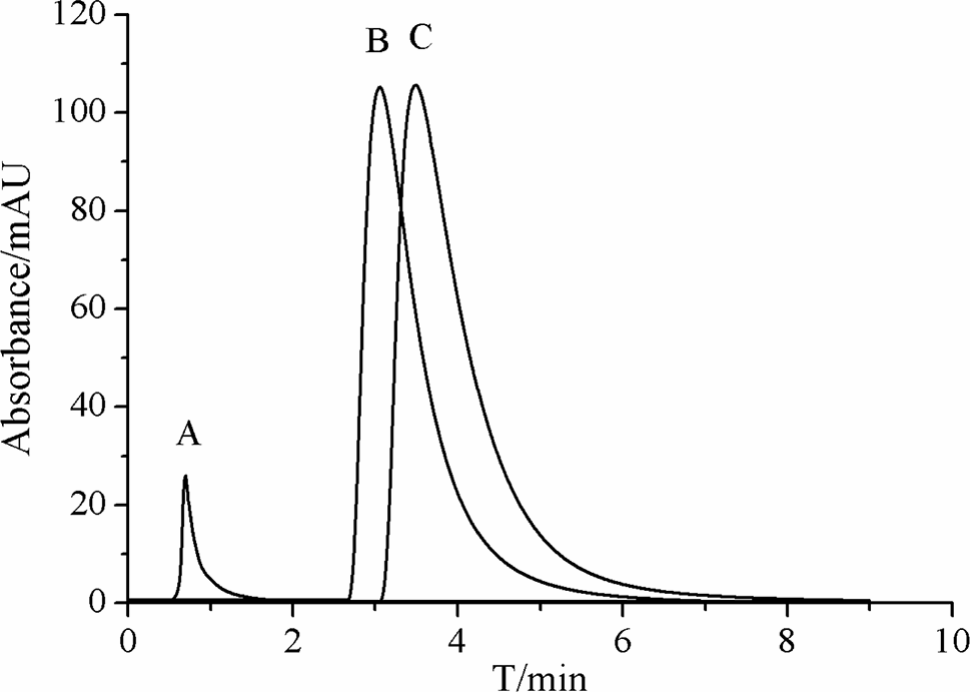
Representative chromatograms of **(A)** sodium nitrite, **(B)** salbutamol, and **(C)** terbutaline on immobilized β_2_-AR column by high-performance liquid chromatography.

Specificity of the immobilized β_2_-AR column was tested by comparing the retention behaviors of salbutamol and terbutaline on the column with those of sodium nitrite. Each of the drugs was injected three times, and the retention time was determined to be 3.2 min for salbutamol, 3.9 min for terbutaline, and 0.7 min for sodium nitrite (**Figure 3**). Under the same conditions, prazosin and terazosin (specific ligands of α_1A_-adrenergic receptor) displayed retention times of 0.8 and 1.0 min. These values were far lower than the retention times of salbutamol and terbutaline and were approximately equal to the retention time of sodium nitrite. This provided a proof of high specificity of the immobilized β_2_-AR and indicated that the immobilized β_2_-AR has the capacity to recognize its specific ligands.

The other characterization of the immobilized β_2_-AR column was the stability investigation. We performed such examination by continuously analyzing the retention times of salbutamol and terbutaline for 3 weeks. The relative standard deviations (RSDs) of their retention times were calculated to be 3.8% and 2.9%, respectively. The chromatogram profiles of the two drugs showed no significant variations in 3 weeks. This result demonstrated that the immobilized β_2_-AR was stable at least in 3 weeks. In that duration, the column remains the bioactivity of recognizing and capturing the unknown ligands of the receptor from complex matrices. It is possible to be utilized for screening β_2_-AR-targeted compounds from *S. grosvenorii*.

### Screening the Bioactive Compounds of LHG

LHG, the fruit of *S. grosvenorii*, is a widely prescribed TCM for the treatment of lung injury, sore throat, and constipation for thousands of years. The chemical constitutions of LHG are cucurbitane triterpene glycosides, glucose, and lipids. A cocktail of references has revealed that the extracted bioactive compounds from *S. grosvenorii* possess good anticancer, antidiabetic, antioxidant, and anti-inflammatory activities (Li et al., 2007; Qi et al., 2008; Lu et al., 2012). The anti-inflammatory effect of the extract was achieved by down-regulating the expression of pro-inflammatory cytokines, iNOS, COX-2, and IL-6 plus up-regulating the inflammation protective genes expression including PARP1 and MAPK9 (Shi et al., 2014). This mechanism has been reported as one of the signaling pathways of β_2_-AR. As a member of superfamily of G-protein-coupled receptors, β_2_-AR has exhibited unambiguous action in curing diseases of respiratory system like asthma. Such curative effect has mainly deepened the initiation of β_2_-AR signaling pathway by forming a receptor–ligand complex. Taken together, we hypothesize that there are β_2_-AR-targeted bioactive compounds in LHG.

**Figure 4:**
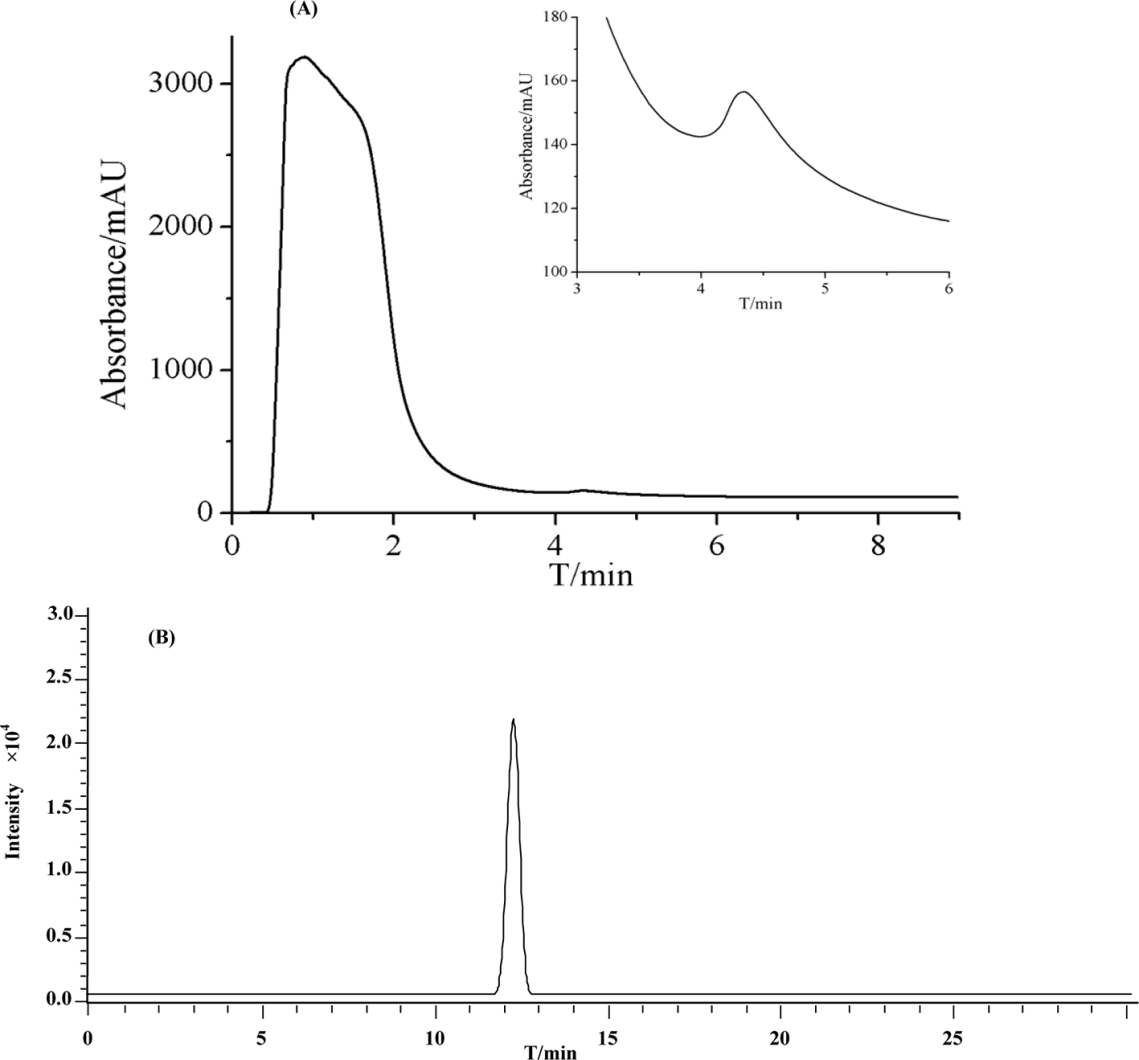
Representative chromatograms of **(A)** LHG extract on the β2-AR column. **(B)** Total ion current of eluted compounds by reversed-phase high-performance liquid chromatography coupled with mass spectrometry.

In the current work, we extracted LHG through a hot refluxing method. Such method has been broadly accepted as a powerful assay for achieving most of the components in LHG. **Figure 4A** displays the chromatogram of LHG aqueous extract on immobilized β_2_-AR column. We found two intensive peaks with retention times of 0.9 and 4.3 min. Regarding the void time of the chromatographic system, we collected the peak at 4.3 min as the compound of interest. We further separated this collection by an Inertsil ODS-3-C_18_ column (5 μm, 4.6 mm × 150 mm) and identified the elution by trap mass spectrometry (**Figure 4B**). The full-scan mass spectrum of this compound showed a deprotonated ion [M−H]^−^ at *m*/*z* 1,285.6 in negative mode. MS/MS result revealed that the father ion yields two daughter ions at *m*/*z* 1,123.6 and *m*/*z* 961.6 by loss of a monomolecular and a bimolecular of C_6_H_10_O_5_. Such mass pattern was in line with MS/MS information of the reference standard of mogroside V. On the basis of these results, we reasoned that the peak at 4.3 min on β_2_-AR column as the bioactive compound binding to the receptor in LHG. Such identification was reasonable since mogroside V has clear action on curing ailments mediated by β_2_-AR.

### Comparison With Other Screening Methods

The screening of bioactive compounds from TCM is in the central part of TCM-guided drug development. Owing to this important role, such research has attracted great attention of researchers in pharmaceutical, chemistry, and biology. Wang et al. (2012) constructed a comprehensive two-dimensional (2D) HPLC system where liposome chromatographic column was utilized as the first dimensional column and the silica monolithic column was regarded as the second dimensional chromatographic column. They applied the system in screening bioactive compounds from *Schisandra chinensis* and identified 14 compounds of the retained peaks. In another report, Wang et al. (2017b) established a comprehensive 2D PC-3 cell membrane chromatography (CMC) system to screen the bioactive compounds of anti-prostate cancer from *Sophorae flavescentis* Ait. and confirmed the structures of five compounds from the herb extract. Li et al. (2013) created an HPLC method taking human albumin as stationary phase to explore the leading compounds by *in vitro* PPB. Wang et al. (2000) utilized immobilized human serum albumin to screen the bioactive compounds from *Artemisia capillaris* and confirmed two strong retention peaks of scoparone and capillarisin. Tong et al. (2014) extracted lipid rafts from brain glioma U251 cells by synthesizing immobilized lipid to screen for anti-tumor bioactive compounds from *Galla chinensis*. Despite their great contribution to promote the development of TCM-guided drugs, the aforementioned reports appear to have certain limitations, like lower separating capacity and specificity. This often makes false-positive results during the utilization of these assays. The exploration of new methodologies still remains open to rapidly recognize, separate, and identify compounds of interest from TCM.

Our group proposed a concept-guided methodology named as receptor chromatography (Li et al., 2015; Wang et al., 2017a; Zeng et al., 2018) to address the limitations of the above assays. In most cases, this method has immobilized the main target like G-protein-coupled receptor of approved drugs onto solid support to achieve column using the immobilized target as stationary phases. Successful applications of the stationary phases have demonstrated high sensitivity, specificity, and stability when it comes to screening the target-binding bioactive compounds. Zhao et al. (2010) screened and identified four bioactive compounds in the aqueous extract of *Coptis chinensis* Franch. by β_2_-AR chromatography. Wang et al. (2017a) analyzed Shuang-Huang-Lian prescription and identified chlorogenic acid as the bioactive compound. This work utilized β_2_-AR chromatographic method for the pursuit of bioactive compounds targeting the receptor from the aqueous extract of LHG. Owing to the high separating capacity, we rapidly recognized mogroside V as β_2_-AR binding bioactive compound. Comparing the methods including immobilized liposome chromatography, we reasoned that the proposed method is possible to enhance the role of chromatographic methods in TCM-guided drug development.

### Mogroside V Exhibited β_2_-AR-Associated Bronchodilator Activities

To prove the bronchodilatory activity of mogroside V, we tested the effects of the compound on the isolated tracheal strips *in vitro*. The potential β_2_-AR-mediating relaxant mechanisms of the compound were also evaluated in the absence and presence of a specific inhibitor. This was performed with the scarification of rats weighing 250–300 g by exsanguination from the abdominal aorta. We collected the tracheal tissues between the larynx and the lungs and immediately immersed them in cold oxygenated Krebs solution (118 mM NaCl, 4.8 mM KCl, 2.5 mM CaCl_2_, 1.2 mM MgSO_4_, 25 mM NaHCO_3_, 1.2 mM KH_2_PO_4_, and 9.8 mM glucose; pH 7.4). After the removal of adhesive tissues, we spirally isolated the tracheal segment with three cartilage rings to assay the tracheal strip reactivity. Such strips were mounted onto two L-shaped stainless-steel holders connected with a force–displacement transducer. The isometric tension was determined in a tissue bath by a PowerLab data acquisition system.

**Figure 5:**
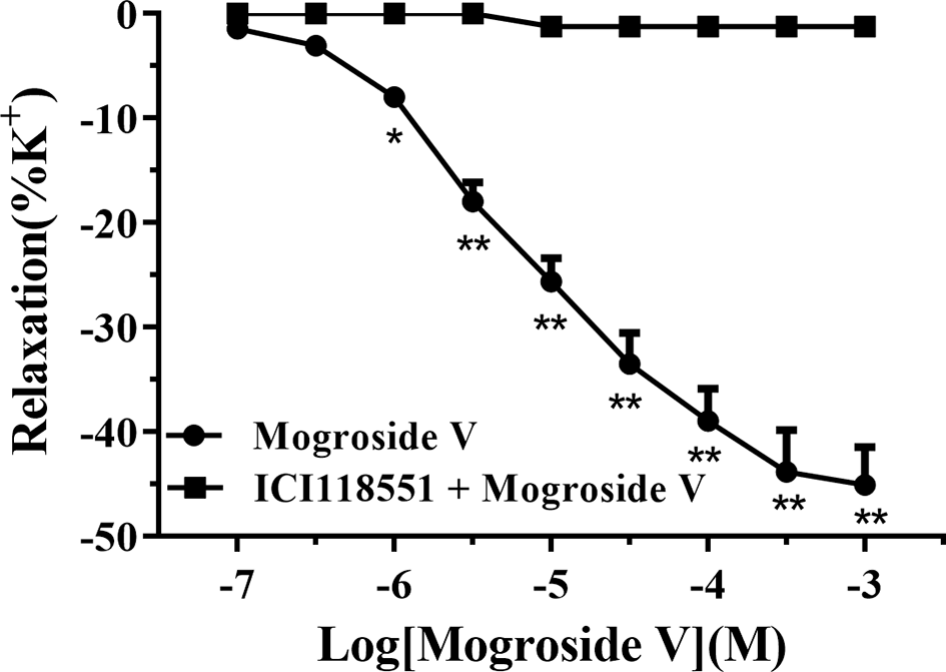
The dose–response curves of mogroside V for the relaxation effect on rat tracheal smooth muscle pre-contracted by high K+ solution (60 mM) in vitro. Each point represents the means ± SEM of each group (n = 6) from two separate experiments. *p < 0.05, **p < 0.01 versus the ICI 118551 treated group.

We aerated the pre-warmed Krebs solution (37°C) with a gas mixture of 95% O_2_ and 5% CO_2_. At initial tests, the tracheal strips with tensions of 1.0-g mass were equilibrated for 60 min. The resulting strips were stimulated with high-potassium (K^+^) solution (63 mM NaCl, 60 mM KCl, 2.5 mM CaCl_2_, 1.2 mM MgSO_4_, 25 mM NaHCO_3_, 1.2 mM KH_2_PO_4_, and 9.8 mM glucose; pH 7.4) to evoke a contractile response in the presence or absence of ICI 118551 (10 μM). When the tensions achieved a plateau value, we cumulatively included mogroside V to induce a relaxant response. **Figure 5** illustrates the influences of mogroside V from 0.1 μM to 1.0 mM on the pre-contracted strip by high-potassium (K^+^) solution. We observed a concentration-dependent manner of mogroside V on the relaxation of tracheal strips in the absence of ICI 118551. Such response disappeared when ICI 118551 was simultaneously applied to the pre-contracted strips. Since ICI 118551 is a specific inhibitor of β_2_-AR, these results suggested that mogroside V is an agonist of the receptor, and the bronchodilator activities of the compound were mediated by β_2_-AR. By these results, we concluded that receptor chromatography is powerful for screening specific ligands of a receptor from traditional Chinese medicine.

### Method Validation for Pharmacokinetics

**Figure 6:**
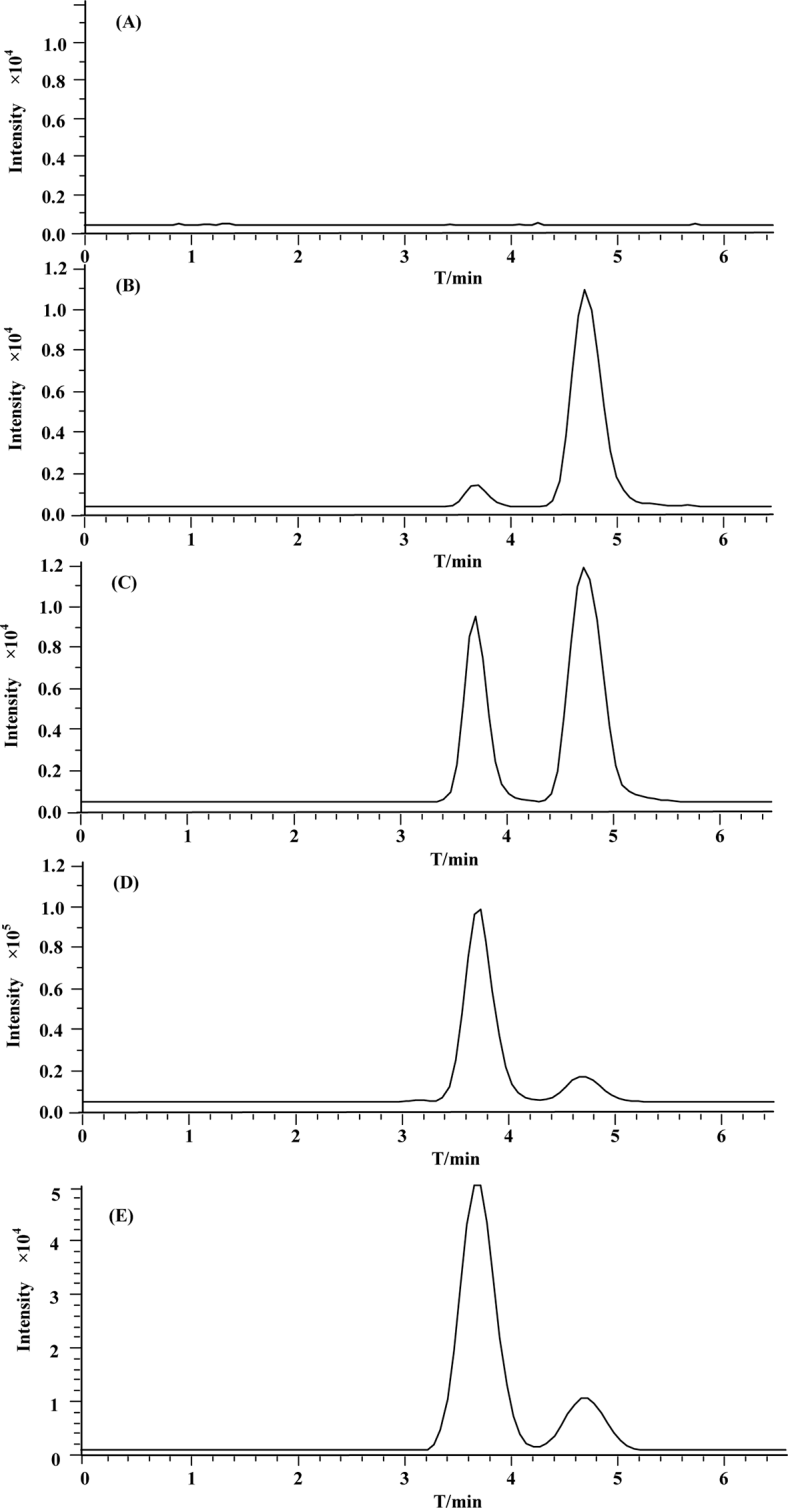
Representative MRM chromatograms of (A) blank rat plasma; (B–D) (QC samples (30 ng/mL (LQC), 500 ng/mL (MQC), and 5,000 ng/mL (HQC), respectively); (E) rat plasma sample at 45 min after 200 mg/kg oral administration of mogroside V.

The MRM chromatograms of blank plasma, QC samples [30 ng/ml (LQC), 500 ng/ml (MQC), and 5,000 ng/ml (HQC)], and plasma collected after 200 mg/kg of oral administration of mogroside V at 45 min are shown in **Figures 6A–E**, respectively. It is obvious that there is no endogenous interference at the retention times of mogroside V (3.6 min) and IS (4.7 min). The calibration curves showed good linearity at the range of 24.4–6,250 ng/ml. The equation of the regression was *y* = 0.0037*x* − 0.0067 (*R* ^2^ = 0.9987), where *y* is the peak area ratio of mogroside V to the IS and *x* is the concentration of mogroside V. Under the proposed conditions, the LLOQ of mogroside V in plasma was determined as 5 ng/mL. The data of intra-day and inter-day accuracy and precision are summarized in **Table 2**. Both the intra-day and inter-day accuracy (RE) and the precision (RSD) were less than 15%. This result was in good accordance with the requirement of bio-assay for pharmacokinetic study by the guideline of Guidance for Industry Bioanalytical Method Validation (U.S. Food and Drug Administration, 2018). The extraction recovery data are summarized in **Table 3** where the recoveries of mogroside V in plasma were all above 85%. The stability data are listed in **Table 4**. This demonstrated that the process of sample preparation generates little variation. Under diverse storing and processing conditions, mogroside V showed a good stability.

**Table 2:** Precision and accuracy of mogroside V in rat plasma (n = 6).

Concentration added (ng/mL)	Intra-day			Inter-day		
	Concentration measured (ng/mL)	RSD %	RE %	Concentration measured (ng/mL)	RSD %	RE %
30	32.3 ± 2.1	6.7	4.2	30.6 ± 7.1	5.5	1.9
500	492.6 ± 8.1	1.6	1.5	493.2 ± 5.6	1.1	1.4
5,000	4,885.5 ± 138.4	2.8	2.3	4,878.4 ± 133.7	2.7	2.4

**Table 3:** Extraction recovery of mogroside V and IS in rat plasma (n = 6).

Compound	Concentration added (ng/mL)	Extraction recovery %	RSD %
	30	89.1 ± 5.6	6.3
Mogroside V	500	92.6 ± 2.1	2.2
	5,000	89.0 ± 1.9	2.2
IS	5,000	92.8 ± 3.3	3.5

**Table 4:** Stability of mogroside V in rat plasma (n = 6).

	Freeze–thaw		Short term		Long term	
Concentration added (ng/ml)	Concentration measured (ng/ml)	Stability (%)	Concentration measured (ng/mL)	Stability (%)	Concentration measured (ng/ml)	Stability (%)
30	27.9 ± 1.7	95.8	28.4 ± 2.5	94.8	28.4 ± 2.9	94.4
500	477.1 ± 9.5	95.4	474.1 ± 4.3	94.8	468.9 ± 8.0	93.8
5,000	4,831.9 ± 9.1	96.6	4,660.1 ± 7.9	93.2	4,607.7 ± 9.0	92.2

Regarding the validated test, we summarized that the proposed HPLC–MS/MS method is sensitive and reliable for the determination of mogroside V in rat plasma. The method has the capacity to analyze the pharmacokinetic behavior of mogroside V in rat plasma after administration of LHG extract.

### Pharmacokinetic Study

**Figure 7:**
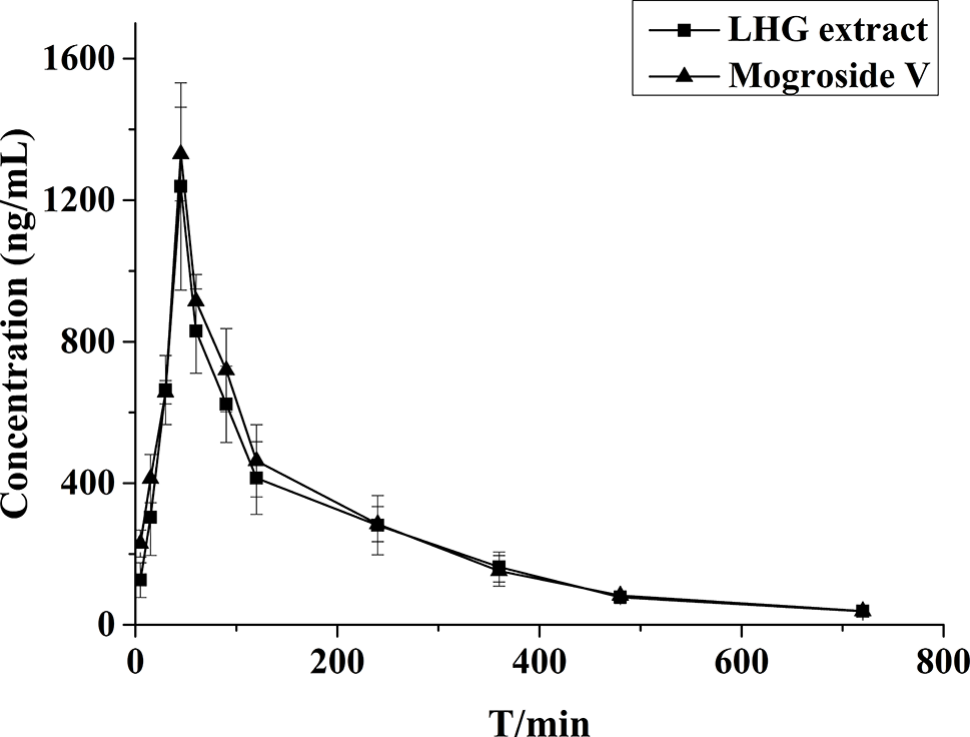
Mean plasma concentration–time profiles of rats after oral administration of mogroside V (200 mg/kg) and LHG extract (12.5 ml/kg).

The validated assay was applied in the pharmacokinetic study of mogroside V and LHG extract in rat plasma after oral administration. The concentration–time curves are described in **Figure 7**. The corresponding pharmacokinetic parameters are summarized in **Table 5**.

**Table 5:** Pharmacokinetic parameters of mogroside V (200 mg/kg) and LHG extract (12.5 ml/kg) orally administered to rats (n = 6 per time point).

Parameters	Unit	Mogroside V	LHG extract
AUC_(0–720)_	ng/L·min	185,549.1 ± 19,169.2	173,693.9 ± 35,301.1
AUC_(0–∞)_	ng/L·min	190,664.8 ± 19,509.2	180,410.2 ± 38,148.6
T_max_	min	45	45
t_1/2z_	min	138.5 ± 13.2	148.3 ± 27.0
C_max_	µg/L	1,330.6 ± 132.2	1,239.2 ± 292.9

By contrastive analysis, we obtained an identical pharmacokinetic behavior between mogroside V and LHG extract after oral administration of desired dose. Both mogroside V and LHG extract showed the time to reach peak plasma concentrations (*C*_max_) at 45 min. The corresponding plasma concentrations were 1,330.6 ± 132.2 and 1,239.2 ± 292.9 ng/ml, respectively. The AUC_(0–720)_ and AUC_(0–∞)_ of mogroside V were 185,549.1 ± 19,169.2 and 190,664.8 ± 19,509.2 ng/L·min, respectively, which changed to 173,693.9 ± 35,301.1 and 180,410.2 ± 38,148.6 ng/L·min when LHG extract was applied. No clear difference was found between the two groups of data, which confirmed the equal dosage of mogroside V during the administration LHG and the compound alone. The ratio of the AUC value was below 120%, providing a proof of rationale time points for blood collection to pursue the pharmacokinetic investigation (Shi et al., 2019). Taking the identical pharmacokinetic behavior into account, we inferred that mogroside V is the representative compounds of LHG for treatment of β_2_-AR-mediating ailments.

A few reports have declared that mogroside V is detectable in rat plasma after tail intravenous injection (1.12 or 2.0 mg/kg) and intraperitoneal administration (1.12 mg/kg) (Zhou et al., 2018), while it disappears after oral administration even though the dose was increased to 5 mg/kg (Luo, 2017). We reasoned that the undetectable results in the case of oral administration are mainly ascribed to the relatively low dose of the compound. Such dose was far lower than the requirement of the compound according to the instruction of LHG in Pharmacopoeia of the People’s Republic of China. On this account, we increased the dose to 200 mg/kg for the pharmacokinetic study. Such dose is in line with the pharmacological study of the compound and has proved to be nontoxic according to the toxicological examination of mogroside V after oral administration by Qin et al. (2006). The study in this work displayed desired pharmacokinetic parameters like slow elimination of mogroside V after oral administration of LHG or the compound alone. This provided a proof of good druggability for mogroside V and indicated that the compound has potential to become a lead compound for further investigation.

## Conclusions

The immobilized β_2_-AR chromatography coupled with HPLC–MS was utilized to screen and identify the bioactive compounds of LHG. Mogroside V was identified as the bioactive compound binding to the β_2_-AR. The druggability of mogroside V was evaluated through the analysis of pharmacokinetic behavior by HPLC–ESI–MS/MS, which illustrated that mogroside V is the main representative of LHG. It demonstrates good druggability for the treatment of β_2_-AR-mediated ailments including asthma. The method of this work is possible to become a powerful strategy for screening bioactive compounds from TCM and pre-evaluating its druggability.

## Data Availability

All datasets generated for this study are included in the manuscript and the supplementary files.

## Ethics Statement

This study was carried out in accordance with the recommendations of Guidelines in Care and Use of Animal under the approval of the Animal Experimentation Ethics Committee at Northwest University.

## Author Contributions

XJ, JL, BS and QiL carried out experiments and wrote the manuscript, XJ, JG and GF analyzed experimental results, ZC, QiaL, XiaZ and JC revised the manuscript, XinZ conceived and designed the experiments.

## Funding

This work was supported by the National Natural Science Foundation of China (Nos. 21705126, 21775119, and 81702832).

## Conflict of Interest Statement

The authors declare that the research was conducted in the absence of any commercial or financial relationships that could be construed as a potential conflict of interest.
